# Research Landscape and Emerging Trends in Herbal Medicine for Pediatric Respiratory Tract Infections: A CiteSpace‐Based Bibliometric Analysis

**DOI:** 10.1002/hsr2.71770

**Published:** 2026-02-09

**Authors:** Chencan Chi, Jianzhong Dang, Fang Zheng

**Affiliations:** ^1^ Department of Pediatrics, Tongji Medical College, Union Hospital Huazhong University of Science and Technology Wuhan Hubei Province China; ^2^ Department of Geriatrics Renmin Hospital of Wuhan University Wuhan Hubei Province China

**Keywords:** bibliometrics, CiteSpace, herbal medicine, pediatrics, respiratory tract infections

## Abstract

**Background and Aims:**

Respiratory tract infections (RTIs) are a common concern in pediatric healthcare. Although extensive literature exists on the use of herbal medicine for pediatric RTIs, no bibliometric analysis has been conducted to date. This study aims to employ bibliometric methods to evaluate the current research landscape in this field and to guide future investigations.

**Methods:**

We systematically retrieved literature on herbal medicine for pediatric RTIs from the Web of Science Core Collection (WoSCC). After screening according to predefined eligibility criteria, 108 articles published between January 2002 and December 2024 were included in the bibliometric analysis using CiteSpace.

**Results:**

Publications in this field have shown fluctuating growth over time. China and Germany emerged as the top contributing countries, with Beijing University of Chinese Medicine and Wolfgang Kamin being the most productive institution and author, respectively. The Cochrane Database of Systematic Reviews published the highest number of papers, while the *Journal of Ethnopharmacology* received the most citations. Research themes primarily focused on the efficacy, safety, and mechanisms of herbal medicine for pediatric RTIs. Key findings highlighted *Pelargonium sidoides* as the most extensively studied herb, acute bronchitis as the predominant condition, respiratory syncytial virus (RSV) as the main pathogen, and the toll‐like receptor 3 (TLR3) signaling pathway as a pivotal therapeutic target. Recent studies have identified network pharmacology and nanoparticle biosynthesis as promising areas for future research in this field.

**Conclusion:**

This study provides a comprehensive overview of herbal medicine for pediatric RTIs. Research in this field is still in its developmental stage. There is a pressing need for enhanced international collaboration to facilitate high‐quality research that can further uncover the therapeutic potential of herbal medicine.

## 1 Introduction

Respiratory tract infections (RTIs) are among the most common pediatric diseases worldwide, contributing to significant morbidity and mortality [1]. However, clinical management of these infections is complicated by challenges such as antibiotic resistance and adverse effects associated with conventional treatments [2]. In this context, herbal medicine has emerged as a promising therapeutic approach for pediatric RTIs. These natural interventions utilize multitargeted mechanisms to alleviate symptoms, modulate immunity, and reduce recurrence, all while maintaining a favorable safety profile [3, 4, 5, 6, 7]. Given the rapid growth of research in this field, a systematic analysis to map its knowledge structure and evolutionary trajectory is urgently needed.

Bibliometric analysis offers a systematic approach to synthesizing the expanding body of literature and mapping the research landscape. Among various bibliometric tools, CiteSpace is distinguished by its exceptional visualization capabilities [8]. Professor Chaomei Chen and his team developed this Java‐based software that converts complex literature data into intuitive knowledge maps. This enables researchers to track disciplinary evolution and identify research trends and frontiers [9, 10]. Although existing reviews have summarized the clinical evidence of herbal medicine for pediatric conditions, a dedicated bibliometric analysis focusing specifically on RTIs is lacking. This gap leaves the knowledge structure, key contributors, and thematic evolution of this field insufficiently understood. To address this, our study employs CiteSpace to conduct the first comprehensive bibliometric analysis in this domain. This approach uniquely allows us to trace the field's development, pinpoint research frontiers, and provide valuable insights to guide future investigations.

## 2 Materials and Methods

### 2.1 Data Source and Search Strategies

The Web of Science Core Collection (WoSCC) was selected as the sole data source for this bibliometric analysis due to its rigorous journal selection, standardized metadata, and high‐quality citation data [11]. These features provide the consistent and homogeneous data essential for accurate citation network analysis and science mapping. This single‐source strategy thus helps maintain analytical integrity while aligning with the study's objective of tracing the evolution of the global research front in this domain. We designed a systematic search strategy to capture the broad, international research landscape of herbal medicine for pediatric RTIs using contemporary scientific terminology. While this approach ensures a focused and consistent data set, it may not exhaustively capture literature framed exclusively within specific traditional systems of medicine that employ distinct terminologies. The search query was formulated as follows: (TS = (herb* OR medicinal plant* OR plant extract* OR herbal medicine OR botanical medicine OR phytotherapy OR phytomedicine OR traditional medicine)) AND (TS = (respiratory tract infection* OR RTI OR respiratory infection*)) AND (TS = (child* OR adolescen* OR minor* OR infant* OR pediatric* OR paediatric*)), where “TS” denotes “Topic Search” and the asterisk (*) serves as a wildcard representing zero or more characters. The initial search covered all records from January 2002 to December 2024, with no restrictions on publication type or language. The complete search strategy is detailed in Table S1.

### 2.2 Inclusion and Exclusion Criteria

The inclusion criteria were as follows: (1) studies focusing on pediatric RTIs; (2) studies in which herbal medicine was the primary intervention; and (3) literature with complete bibliographic records, including title, abstract, authors, publication year, institution, country, keywords, journal, and references. The exclusion criteria were: (1) studies that did not address the use of herbal medicine in the context of pediatric RTIs; (2) literature with incomplete bibliographic records; and (3) literature types such as conference proceedings, book chapters, editorials, clinical guidelines, expert consensus documents, early access articles, and retracted publications.

### 2.3 Data Collection and Processing

We exported the selected literature as plain text files containing full records and cited references, renaming the files using a format compatible with CiteSpace (e.g., download_xx.txt). Duplicates were removed both automatically using CiteSpace and manually to ensure thoroughness. The extracted data then underwent a comprehensive cleaning process, which involved removing extraneous text, correcting spelling errors, and standardizing institutions, authors, journals, and keywords. This standardization was achieved by employing a thesaurus file to merge identical entries and synonyms. For example, “Institute of Basic Research in Clinical Medicine” and “Institute of Chinese Materia Medica” were unified under “China Academy of Chinese Medical Sciences,” while “respiratory system infection” and “vitamin C” were mapped to “respiratory tract infections” and “ascorbic acid,” respectively. To ensure accuracy and consistency, two researchers independently conducted the literature screening and data cleaning processes. Any discrepancies were resolved through discussion until a consensus was reached.

### 2.4 Data Analysis and Visualization

We used Microsoft Excel 2021 to analyze the quantity and temporal trends of research objectives in the literature, generating relevant charts in the process. Concurrently, we employed CiteSpace 6.4.R1 to perform a visual analysis of the refined literature data sets and generate knowledge maps. The parameters of CiteSpace were configured as follows: (1) time slicing: the time span was set from January 2002 to December 2024, with annual intervals; (2) node types: the selected node types included country, institution, author, cited journal, reference, and keyword; (3) selection criteria: the g‐index algorithm with *k* = 25 was utilized; (4) pruning: for keywords, the Pathfinder, Pruning Sliced Networks, and Pruning the Merged Network functions were applied to simplify the network map and highlight key nodes and their connections; (5) cluster analysis: the K‐means algorithm was used to cluster the keyword co‐occurrence network, followed by log‐likelihood ratio (LLR) tests for cluster annotation. All other parameters were set to their default values.

The parameter configurations in CiteSpace were deliberately chosen to enhance the robustness and interpretability of the analysis. Using a 1‐year time slicing approach allows for a detailed examination of thematic evolution within the field. The g‐index, with a parameter of *k* = 25, was applied to include high‐frequency core nodes as well as significant mid‐ and low‐frequency nodes, thereby balancing representativeness and comprehensiveness. For clustering, the K‐means algorithm was employed due to its effectiveness in handling large‐scale text data and uncovering underlying thematic patterns from keyword co‐occurrence networks.

## 3 Results

### 3.1 Analysis of Annual Publication Trends

**Figure 1:**
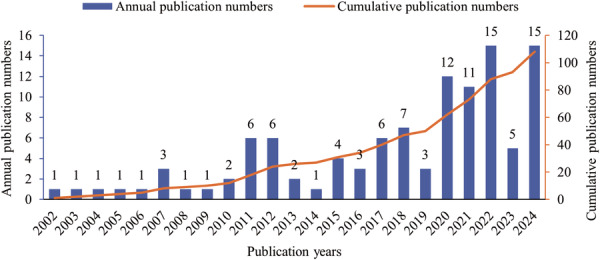
Trend chart of annual number of publications and cumulative number of publications from 2002 to 2024.

According to our search criteria, 108 articles on herbal medicine for pediatric RTIs were identified, comprising 75 original studies and 33 reviews. As illustrated in Figure 1, publication trends can be divided into three phases. The first phase (2002–2014) was an exploratory period with only 27 publications in total, characterized by low and fluctuating output. The second phase (2015–2019) showed steady growth, with 23 articles published over 5 years, reflecting increasing academic interest. The third phase (2020–2024) experienced rapid expansion, with 58 articles published within 5 years. Publication numbers first exceeded 10 in 2020 and peaked at 15 in both 2022 and 2024. Despite a slight dip in 2023, overall growth remains strong, indicating a significant rise in research interest in recent years.

### 3.2 Analysis of Countries and Institutions

**Figure 2:**
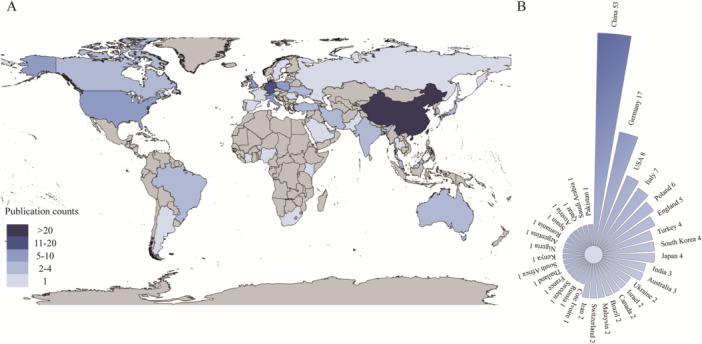
(A) Geographical distribution of global research output. (B) Contributions from different countries.

**Figure 3:**
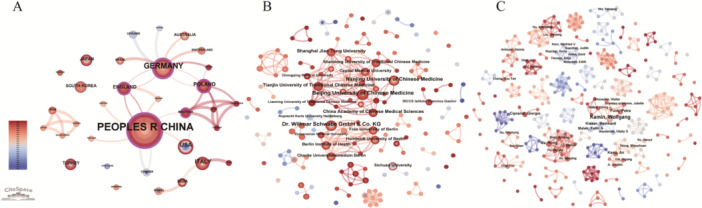
Cooperative network analysis. (A) Countries, (B) institutions, and (C) authors.

We identified 154 institutions across 33 countries contributing to this field. Figure 2A illustrates a regionally concentrated productivity distribution, with Asia and Europe serving as the primary research hubs. Figure 2B presents national contributions, with China leading at 53 publications, followed by Germany (17), the USA (8), Italy (7), and Poland (6) which constitute the core research forces. Table 1 ranks the top 5 countries by centrality, highlighting Germany (0.33) as playing a pivotal role in collaboration, followed by China (0.24), England (0.15), Poland (0.14), and Malaysia (0.12). The national cooperation network (Figure 3A) reveals a limited scope of international collaboration. Germany maintains close partnerships with Poland and England, while China primarily collaborates with England and the United States.

**Table 1:** Top 5 countries by centrality.

Rank	Country	Centrality
1	Germany	0.33
2	China	0.24
3	England	0.15
4	Poland	0.14
5	Malaysia	0.12

Table 2 lists the top 5 research institutions by publication volume. This highlights the leading role of academic institutions and pharmaceutical companies, particularly Chinese universities, in researching herbal medicine for pediatric RTIs. Table 3 presents the top 5 research institutions by centrality. Beijing University of Chinese Medicine ranks highest with a centrality score of 0.02, and also leads in publication volume with eight publications, reflecting its dual role as both a research leader and a collaboration hub. Figure 3B illustrates limited institutional collaboration, characterized by a dual‐core structure centered on Beijing University of Chinese Medicine and Dr. Willmar Schwabe GmbH & Co. KG. The former primarily partners with domestic traditional Chinese medicine (TCM) institutions, while the latter focuses on collaborations within Germany.

**Table 2:** Top 5 organizations by publications.

Rank	Institution	Publications
1	Beijing University of Chinese Medicine	8
2	Dr. Willmar Schwabe GmbH & Co. KG	8
3	Nanjing University of Chinese Medicine	6
4	Shanghai Jiao Tong University	5
5	Tianjin University of Traditional Chinese Medicine	5

**Table 3:** Top 5 organizations by centrality.

Rank	Institution	Centrality
1	Beijing University of Chinese Medicine	0.02
2	Nanjing University of Chinese Medicine	0.01
3	Shanghai Jiao Tong University	0.01
4	Tianjin University of Traditional Chinese Medicine	0.01
5	Capital Medical University	0.01

### 3.3 Analysis of Authors

A total of 334 scholars worldwide have made significant contributions to the research on herbal medicine for pediatric RTIs. As shown in Table 4, the ten most prolific authors include Wolfgang Kamin from Evangel Hospital in Hamm, Germany, who leads the field with five publications. Following closely are Meinhard Kieser, Fathi A. Malek, An Kang, Petra Funk, Rong Ma, and Giorgio Ciprandi, each with three publications. Other key contributors include Gerd Antes, Judith Guenther, and Winfried V. Kern, each with two publications.

**Table 4:** Top 10 authors ranked by publication volume.

Rank	Author	Country	Institution	Begin year	Publications
1	Wolfgang Kamin	Germany	Evangel Hospital	2010	5
2	Meinhard Kieser	Germany	Heidelberg University	2010	3
3	Fathi A. Malek	Germany	Dr. Willmar Schwabe GmbH & Co. KG	2010	3
4	An Kang	China	Nanjing University of Chinese Medicine	2015	3
5	Petra Funk	Germany	D.r Willmar Schwabe GmbH & Co. KG	2018	3
6	Rong Ma	China	Tianjin University of Traditional Chinese Medicine	2018	3
7	Giorgio Ciprandi	Italy	Casa di Cura Villa Montallegro	2021	3
8	Gerd Antes	Germany	University Medical Center Freiburg	2008	2
9	Judith Guenther	Germany	Pharmafacts GmbH Research & Consulting Drug Care	2008	2
10	Winfried V. Kern	Germany	University Medical Center Freiburg	2008	2

Figure 3C illustrates a decentralized author cooperation network in the field of herbal medicine for pediatric RTIs. This network is characterized primarily by small‐scale teams and limited intergroup collaborations, with most interactions occurring within teams rather than between different groups. However, emerging teams led by scholars such as Wolfgang Kamin, An Kang, and Rong Ma exhibit stronger internal collaboration, higher productivity, and denser network connections. Although collaboration in this field remains underdeveloped, these groups demonstrate promising potential to advance research progress.

### 3.4 Journal Distribution

The analyzed literature was published across 80 academic journals. Table 5 lists the top 10 journals by publication volume, which collectively published 36 documents, accounting for 33.33% of the total output. The 2024 Journal Citation Reports (JCR) and Impact Factors (IF) were obtained from the Web of Science and served as key indicators of journal impact. Half of the top 10 journals were ranked in the Q1 JCR division, with IFs primarily ranging from 1.4 to 5.5. The most productive journal was the Cochrane Database of Systematic Reviews (8 publications, IF = 9.4), followed by *Frontiers in Pharmacology* (7 publications, IF = 4.8).

**Table 5:** Top 10 journals with the most publications.

Rank	Journal	Country	Count	Percentage (%)	IF (2024)	JCR
1	*Cochrane Database of Systematic Reviews*	England	8	7.41	9.4	Q1
2	*Frontiers in Pharmacology*	Switzerland	7	6.48	4.8	Q1
3	*Journal of Ethnopharmacology*	Holland	4	3.70	5.4	Q1
4	*Medicine*	USA	4	3.70	1.4	Q2
5	*Evidence‐Based Complementary and Alternative Medicine*	England	3	2.78	—	—
6	*African Journal of Traditional, Complementary and Alternative Medicines*	Nigeria	2	1.85	—	—
7	*American Journal of Chinese Medicine*	USA	2	1.85	5.5	Q1
8	*Biomedicine & Pharmacotherapy*	France	2	1.85	7.5	Q1
9	*Combinatorial Chemistry & High Throughput Screening*	The United Arab Emirates	2	1.85	1.7	Q3/Q4
10	*Current Medical Research and Opinion*	England	2	1.85	2.2	Q2/Q3

Table 6 lists 10 most cited journals, most of which are classified in the Q1 JCR division, indicating their high academic quality. The *Journal of Ethnopharmacology* had the highest number of citations (*n* = 46, IF = 5.4), followed by *Phytomedicine* (*n* = 39, IF = 8.3). The *Annals of Internal Medicine* exhibited the highest centrality (0.24), followed by the *Cochrane Database of Systematic Reviews* (0.20). Notably, most of these highly cited journals are based in England and the United States.

**Table 6:** Top 10 journals in terms of co‐citation counts.

Rank	Journal	Country	Co‐citation count	Centrality	Begin year	IF (2024)	JCR
1	*Journal of Ethnopharmacology*	Holland	46	0.06	2002	5.4	Q1
2	*Phytomedicine*	Germany	39	0.07	2003	8.3	Q1
3	*Cochrane Database of Systematic Reviews*	England	36	0.20	2007	9.4	Q1
4	*Lancet*	England	35	0.04	2004	88.5	Q1
5	*Phytotherapy Research*	England	33	0.05	2002	6.3	Q1
6	*Antiviral Research*	Holland	31	0.17	2002	4.0	Q1
7	*Annals of Internal Medicine*	USA	27	0.24	2002	15.2	Q1
8	*PLOS One*	USA	25	0.04	2018	2.6	Q2
9	*Frontiers in Pharmacology*	Switzerland	23	0.01	2020	4.8	Q1
10	*New England Journal of Medicine*	USA	23	0.05	2002	78.5	Q1

**Figure 4:**
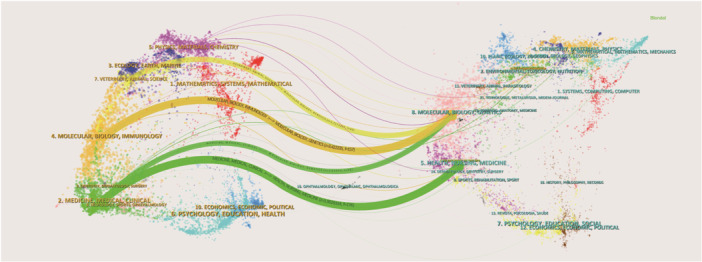
The dual‐map overlay of journals related to herbal medicine for pediatric RTIs.

The dual‐map overlay of journals offers a distinctive perspective on the distribution of academic literature, citation patterns, and the evolution of research focus [12]. Figure 4 illustrates this overlay of journals related to herbal medicine for pediatric RTIs. The left side of the map displays citing journals, while the right side presents cited journals. Curves indicate citation links, with line width reflecting citation frequency measured by *Z*‐score. Most publications appear in journals related to veterinary/animal/science, molecular/biology/immunology, and medicine/medical/clinical fields, whereas the majority of cited publications are found in molecular/biology/genetics and health/nursing/medicine journals. The map outlines four main citation paths: articles in veterinary/animal/science and molecular/biology/immunology journals partially cite molecular/biology/genetics journals; articles in medicine/medical/clinical journals predominantly cite health/nursing/medicine journals, with some partial citation of molecular/biology/genetics journals.

### 3.5 Analysis of Co‐Cited References and Citation Bursts

Literature co‐citation analysis is an effective method for identifying high‐impact publications that often form the theoretical foundation of a research field and influence subsequent studies [13, 14]. As shown in Table 7, the top 10 most‐cited articles include 9 original research papers and 1 review article, half of which are clinical trials. This distribution underscores the field's emphasis on clinical research concerning herbal medicine for pediatric RTIs. Notably, seven of these highly cited publications focus on *Pelargonium sidoides* root extract EPs 7630, highlighting its importance in current research. The studies by Matthys and Leydesdorff [15] and Haidvogl and Heger [16] had the highest co‐citation counts. Both demonstrated that EPs 7630 effectively alleviates bronchitis symptoms, achieves high patient satisfaction, and has a favorable safety profile. These findings provide strong evidence for its use in treating pediatric acute bronchitis.

**Table 7:** The top 10 co‐cited articles.

Rank	First author	Title	Document type	Year	Journal	Co‐citation count
1	H. Matthys	Treatment of acute bronchitis with a liquid herbal drug preparation from *Pelargonium sidoides* (EPs 7630): A randomized, double‐blind, placebo‐controlled, multicentre study	Article	2007	*Current Medical Research and Opinion*	5
2	M. Haidvogl	Treatment effect and safety of EPs® 7630‐solution in acute bronchitis in childhood: Report of a multicentre observational study	Article	2007	*Phytomedicine*	5
3	D. Careddu	*Pelargonium sidoides* extract EPs 7630: A review of its clinical efficacy and safety for treating acute respiratory tract infections in children	Review	2018	*International Journal of General Medicine*	4
4	J. Papies	Antiviral and immunomodulatory effects of *Pelargonium sidoides* DC. root extract EPs® 7630 in SARS‐CoV‐2‐infected human lung cells	Article	2021	*Frontiers in Pharmacology*	4
5	V. V. Berezhnoi	Clinical efficacy and safety of liquid *Pelargonium sidoides* preparation (EPs 7630) in children with acute non‐streptococcal tonsillopharyngitis	Article	2016	*Journal of Comprehensive Pediatrics*	4
6	D. Martin	Reduced antibiotic use after initial treatment of acute respiratory infections with phytopharmaceuticals—A retrospective cohort study	Article	2020	*Postgraduate Medicine*	4
7	A. Conrad	Extract of *Pelargonium sidoides* (EPs® 7630) improves phagocytosis, oxidative burst, and intracellular killing of human peripheral blood phagocytes in vitro	Article	2007	*Phytomedicine*	4
8	A. Conrad	Extract of *Pelargonium sidoides* (EPs® 7630) inhibits the interactions of group A—streptococci and host epithelia in vitro	Article	2007	*Phytomedicine*	4
9	B. P. Parrett	Treatment of the common cold with unrefined echinacea. A randomized, double‐blind, placebo‐controlled trial	Article	2002	*Annals of Internal Medicine*	3
10	H. A. Cohen	Effectiveness of an herbal preparation containing echinacea, propolis, and vitamin C in preventing respiratory tract infections in children	Article	2004	*Archives of Pediatrics & Adolescent Medicine*	3

**Figure 5:**
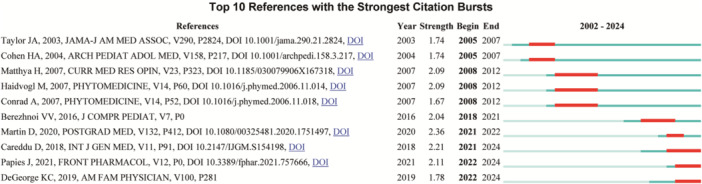
Top 10 references with the strongest citation bursts.

Through citation burst analysis (Figure 5), we identified ten references exhibiting the strongest citation bursts. The retrospective cohort study conducted by Martin et al. [17] in *Postgraduate Medicine* demonstrated the highest citation burst strength of 2.36. This large‐scale clinical study revealed that early treatment with specific phytopharmaceuticals for acute respiratory tract infections significantly reduces the need for antibiotics and shortens the duration of sick leave, thereby providing a viable strategy to combat antibiotic misuse. Furthermore, our analysis indicated sustained citation growth from 2021 to 2024 for studies by Careddu and colleagues, Papies and colleagues, and DeGeorge and colleagues, highlighting their continuing academic influence in this research area in recent years.

### 3.6 Keyword Analysis

**Figure 6:**
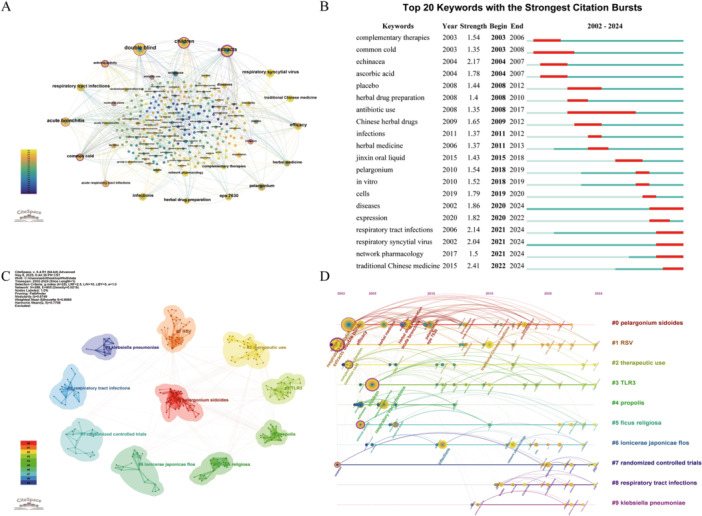
Visualization analysis of keywords. (A) Co‐occurrence network; (B) top 20 keywords with the strongest citation bursts; (C) cluster map; (D) timeline view.

Keyword co‐occurrence network analysis identifies research hotspots and trends by examining the frequency and relationships of keywords in academic literature. This method visually maps the connections between knowledge units, revealing the structural framework of a field [18]. Figure 6A illustrates this network. The top 10 keywords by frequency are “double‐blind,” “extracts,” “children,” “acute bronchitis,” “RTIs,” “respiratory syncytial virus (RSV),” “efficacy,” “infections,” “EPs 7630,” and “herbal drug preparation,” which reflect core research themes in this field.

Burst keywords are terms that experience significant increases in usage over specific periods, helping to identify research hotspots and track the evolution of topics [19, 20]. Figure 6B displays the top 20 keywords ranked by burst duration, where blue segments represent research periods and red segments indicate burst durations. “Complementary therapies” and “common cold” were among the earliest burst keywords, reflecting the initial research focus on alternative treatments for the common cold [21, 22]. “Antibiotic use” exhibited the longest burst duration, indicating sustained scholarly interest, while “TCM” showed the highest burst intensity (2.41), underscoring its particular significance in this research domain [23, 24, 25].

Cluster analysis reveals the knowledge structure and key research themes within a field by quantifying keyword similarities and co‐occurrences [26]. We employed K‐means clustering to classify keywords and labeled the clusters using LLR tests, as shown in Figure 6C. Cluster validity was quantitatively assessed using Modularity (*Q*) and the Mean Silhouette Score (*S*). A *Q* value > 0.3 indicates a significant cluster structure, while an *S* value above 0.7 signifies strong internal consistency and clear separation between clusters. Our clustering results were robust, with a *Q* value of 0.6749 and an *S* value of 0.8985, demonstrating a clear and convincing cluster structure. The ten major clusters identified were: #0 *Pelargonium sidoides*, #1 RSV, #2 therapeutic use, #3 toll‐like receptor 3 (TLR3), #4 propolis, #5 *Ficus religiosa*, #6 *Lonicerae japonicae flos*, #7 randomized controlled trials (RCTs), #8 RTIs, and #9 *Klebsiella pneumoniae*.

We constructed a keyword timeline (Figure 6D) to clarify the evolution of research hotspots, with years represented on the horizontal axis and cluster numbers on the vertical axis. Nodes denote prominent keywords, while connecting lines depict their temporal progression. Our analysis revealed that clusters #1, #2, #3, #5, and #7 demonstrated significant continuity, indicating sustained research interest. Consistent research focuses included the efficacy, safety, and mechanisms of herbal medicine for pediatric RTIs, investigated in various forms such as extracts, decoctions, patches, and teas. Key mechanistic emphases encompassed antiviral, antibacterial, anti‐inflammatory, and immunomodulatory effects.

## 4 Discussion

Our bibliometric analysis of 108 articles published over 23 years reveals that the research landscape concerning herbal medicine for pediatric RTIs is rapidly evolving, yet still in its early stages. Although the volume of literature is increasing, it reflects a field that remains developmental. The most notable finding is the significant surge in publications over the past 5 years, which has accelerated the field's growth. This trend corresponds with the global expansion of complementary and alternative medicine and may have been further intensified by the increased focus on respiratory health and antimicrobial strategies during the COVID‐19 pandemic [27, 28, 29, 30]. By systematically mapping this growth, our analysis addresses the urgent need to understand the knowledge structure and evolutionary trajectory of this promising field.

The global distribution of research was highly concentrated, with the top 5 most productive countries accounting for 81.48% of the total output. China and Germany emerged as the primary drivers. However, our network analysis revealed a significant limitation: both national and institutional collaborations remain limited, with partnerships largely confined within domestic borders. Notably, collaboration between leading Chinese and German institutions is particularly weak. This insular pattern of collaboration suggests that the field is progressing through parallel, regionally focused efforts rather than through integrated global synergy. Therefore, it is imperative to strategically prioritize the establishment of multinational research networks and resource‐sharing frameworks to enhance innovation capacity and ensure the global relevance of findings.

The author analysis revealed varying distributions of research influence across countries. While China led in total output with a broad but less concentrated author base, Germany excelled in developing productive core teams. Kamin from Germany emerged as a leading researcher, primarily due to his team's work on the efficacy and safety of EPs 7630 in pediatric RTIs [31, 32]. Their rigorous studies established the clinical value of EPs 7630 in treating pediatric acute bronchitis, providing a foundation for standardized application of herbal medicine in this field [33, 34, 35, 36]. This disparity in research quality underscores the need for Chinese scholars to cultivate innovative core research teams while maintaining their output advantages.

Core journals play a pivotal role in disseminating novel research findings and reflecting disciplinary trends. They enable researchers to track the evolution of their fields and identify suitable publication venues. Our analysis of core journals revealed that most of the top 10 were based in England or the United States. Despite China's significant research contributions, Asian journals were underrepresented among the leading publishers, highlighting the need to enhance their global standing. Moreover, only a 20% overlap was observed between high‐volume and high‐citation journals, indicating that publication volume and citation frequency represent distinct dimensions of scholarly impact. Thus, high productivity is not a reliable predictor of profound academic influence.

Keyword co‐occurrence and cluster analyses revealed a research landscape initially focused on specific conditions and interventions, most notably acute bronchitis and EPs 7630, supported by multiple RCTs and meta‐analyses [37, 38, 39]. However, our bibliometric mapping identified a significant concentration of studies. The predominance of research on EPs 7630, primarily conducted by German teams, accurately reflects the current evidence base. It also suggests a research network and citation structure potentially influenced by strong geographic and commercial interests. This concentration highlights the risk of regional bias in the evidence base and underscores the need for more geographically diverse and independent research to validate and generalize the findings.

In parallel, RSV has been recognized as a prominent and distinct pathogen within the research community, reflecting its significant clinical burden in pediatrics. The field's focus has shifted from merely identifying clinical candidates to conducting in‐depth mechanistic investigations, which are now elucidating key pathological pathways such as TLR3‐mediated inflammation and oxidative stress [40, 41, 42]. Compounds such as andrographolide sulfonate, resveratrol, and Jinxin oral liquid have demonstrated activity within these frameworks [43, 44, 45]. This clear progression from clinical observation to exploring underlying biological mechanisms represents an important trend toward deeper research insights.

The burst detection algorithm, which tracks shifts in research trends and short‐term spikes in citation or keyword popularity, effectively identifies emerging research areas [46, 47]. In this study, the algorithm initially detected “complementary therapies” and “common cold” as keyword bursts in 2003. Subsequently, the research focus expanded from these broad categories to specific herbal species and preparations, as reflected by emerging keywords like “echinacea,” “ascorbic acid,” “Chinese herbal drugs,” “Jinxin oral liquid,” and “*Pelargonium*.” Interestingly, the keyword “antibiotic use” exhibited the longest burst duration, highlighting global concerns about antibiotic resistance in children and positioning herbal medicine as a potential solution. The rising interest in keywords such as “placebo,” “in vitro,” and “cells” indicated a shift from clinical observation toward a more diverse research approach that incorporates basic medical experiments. Since 2021, sustained bursts of keywords like “RSV,” “network pharmacology,” and “TCM” have reflected a growing academic focus on applying network pharmacology to explore TCM for treating pediatric RSV infections [48, 49].

Timeline visualization can further elucidate the evolution of research themes. This analysis revealed a clear trajectory characterized by the diversification of herbal sources, an expanded range of pediatric RTIs under investigation, and a progression toward more in‐depth mechanistic studies. Within this evolving landscape, network pharmacology has emerged as a promising future direction. It offers transformative methodologies particularly well‐suited to addressing pediatric‐specific challenges. This approach provides novel, systems‐level perspectives for decoding the polypharmacological effects of herbal therapies in pediatric RTIs. By integrating multiomics data and computational modeling, network pharmacology can unravel the complex interactions among herbal compounds, biological targets, and disease pathways [50]. For pediatric safety, this methodology is especially valuable, it can predict potential off‐target effects on key developmental pathways, thereby identifying herbs or compounds that should be used with caution in children. Furthermore, by constructing age‐specific protein interaction networks, it could help explain differential efficacy and optimal dosing of herbal therapies across various pediatric age groups, moving beyond simple weight‐based adjustments. This approach would address the inherent complexity of herbal therapies and may establish the theoretical foundations for their standardized application.

Meanwhile, the biosynthesis of nanoparticles using plant extracts has gained attention as a potential treatment approach for pediatric RTIs. This technology enables green synthesis by integrating plant active components into nanomaterials, potentially endowing the resulting nanoparticles with unique biomedical properties [51]. Preliminary studies suggest that they are effective against multidrug‐resistant respiratory pathogens and can penetrate bacterial biofilms due to their size and surface characteristics, while maintaining the safety profile of natural medicines and the high efficiency of nanomaterials [52, 53]. Furthermore, the composite system offers favorable targeting and slow‐release capabilities, thereby enhancing therapeutic precision [54]. In pediatrics, these features are particularly promising for improving dosing accuracy and safety. For example, nanoparticle‐based delivery systems can be designed to mask the bitter taste of herbal drugs, a critical factor in enhancing medication adherence among children. More importantly, their targeting capability could allow for lower and more precise doses, minimizing systemic exposure and reducing the risk of toxicity to developing organs. However, current research is largely confined to in vitro experiments and lacks systematic in vivo efficacy validation, long‐term toxicity data, and clearly defined metabolic pathways [55, 56]. Clinical translation remains at an early stage [57]. Future efforts should prioritize standardized preclinical trials, comprehensive safety evaluation systems, in‐depth mechanistic exploration, and multicenter clinical studies to address these gaps.

## 5 Limitations

This study has several key limitations:

- Database restriction: The exclusive use of WoSCC ensures methodological consistency but introduces inherent language and geographic biases. Consequently, our findings primarily reflect the international English‐language research landscape and likely underrepresent significant contributions published in regional databases or in languages other than English.
- Limited data set size: Although the analytical corpus is representative of this specialized field, its relatively small size limits the statistical robustness of trend analysis and the detection of subtle patterns. This limitation is inherent to the field's current stage of development.
- Citation lag: The use of citation‐based metrics is subject to a time lag. Recent, high‐quality publications may not have had sufficient time to accumulate citations, which could skew the analysis toward older works and underrepresent emerging research trends.
- Potential publication bias: The published literature is biased toward well‐funded and commercialized products, which may lead to the underrepresentation of noncommercial traditional medicines. Furthermore, the prevalence of positive results in published studies could distort the perceived research focus.
- Terminological focus: Our search strategy centered on core keywords related to “herbal medicine” and may have excluded studies framed exclusively within specific traditional medical systems. As a result, the knowledge structure and trends identified in this study primarily reflect mainstream international scientific discourse and may not adequately capture the unique research paradigms and contributions of these traditional domains.

## 6 Conclusion

Our bibliometric analysis reveals a clear and growing interest in researching herbal medicine for treating pediatric RTIs, as evidenced by the annual increase in publications on the topic. China and Germany are the leading contributors in this field, although international collaboration is still limited. Current research is concentrated on evaluating clinical efficacy and safety, as well as elucidating mechanisms of action, including antiviral, antibacterial, anti‐inflammatory, and immunomodulatory effects. The research frontier is expanding toward advanced pharmacological approaches, with network pharmacology and nanoparticle applications emerging as promising future directions. Furthermore, the development of standardized clinical protocols and the strengthening of translational research are likely to become critical areas of focus. These findings offer valuable insights for clinicians and researchers by outlining current research priorities and suggesting potential avenues for future work.

## Author Contributions

**Chencan Chi:** conceptualization, data curation, writing – original draft, software, methodology, formal analysis, visualization, investigation. **Jianzhong Dang:** data curation, writing – review and editing, software, validation, investigation. **Fang Zheng:** conceptualization, writing – review and editing, supervision, methodology, project administration, resources. All authors have read and approved the final version of the manuscript. Fang Zheng had full access to all of the data in this study and takes complete responsibility for the integrity of the data and the accuracy of the data analysis.

## Disclosure

The lead author Fang Zheng affirms that this manuscript is an honest, accurate, and transparent account of the study being reported; that no important aspects of the study have been omitted; and that any discrepancies from the study as planned (and, if relevant, registered) have been explained.

## Conflicts of Interest

The authors declare no conflicts of interest.

## Supporting information


**Table S1:** Search strategy for the Web of Science Core Collection.

## Data Availability

The original data for this study are publicly available from the Web of Science Core Collection, while the derived data sets analyzed are available from the corresponding author upon reasonable request.
