# Supplementary material for: Research Landscape and Emerging Trends in Herbal Medicine for Pediatric Respiratory Tract Infections: A CiteSpace‐Based Bibliometric Analysis
Source: Health Sci Rep. 2026 Feb 9;9(2):e71770. doi: 10.1002/hsr2.71770 (PMC12886189; doi:10.1002/hsr2.71770)
Supplement: Supplementary file 1 — Table S1: Search strategy for the Web of Science Core Collection. [file HSR2-9-e71770-s001.doc]

**TABLE S1** Search strategy for the Web of Science Core Collection.

| Step | Search Query |
| --- | --- |
| #1 | TS=(herb* OR medicinal plant* OR plant extract* OR herbal medicine OR botanical medicine OR phytotherapy OR phytomedicine OR traditional medicine) |
| #2 | TS=(respiratory tract infection* OR RTI OR respiratory infection*) |
| #3 | TS=(child* OR adolescen* OR minor* OR infant* OR pediatric* OR paediatric*) |
| #4 | #1 AND #2 AND #3 |
| #5 | Filters: Publication years (2002–2024) |
| #6 | Initial search results: 274 records |
